# The N-terminal domain of the prion protein is required and sufficient for liquid–liquid phase separation: A crucial role of the Aβ-binding domain

**DOI:** 10.1016/j.jbc.2021.100860

**Published:** 2021-06-06

**Authors:** Janine Kamps, Yu-Hsuan Lin, Rosario Oliva, Verian Bader, Roland Winter, Konstanze F. Winklhofer, Jörg Tatzelt

**Affiliations:** 1 Department of Biochemistry of Neurodegenerative Diseases, Institute of Biochemistry and Pathobiochemistry, Ruhr University Bochum, Bochum, Germany; 2 Cluster of Excellence RESOLV, Ruhr University Bochum, Bochum, Germany; 3 Division of Physical Chemistry I - Biophysical Chemistry, Faculty of Chemistry and Chemical Biology, TU Dortmund University, Dortmund, Germany; 4 Department of Molecular Cell Biology, Institute of Biochemistry and Pathobiochemistry, Ruhr University Bochum, Bochum, Germany

**Keywords:** liquid–liquid phase separation, neurodegenerative disease, prion protein, prion disease, protein self-assembly, aggregation, intrinsically disordered protein, DLS, dynamic light scattering, FRAP, fluorescence recovery after photobleaching, LLPS, liquid–liquid phase separation, MBP, maltose-binding protein, PrP, prion protein, PrP^C^, cellular prion protein, TEV, tobacco etch virus

## Abstract

Formation of biomolecular condensates through liquid–liquid phase separation (LLPS) has been described for several pathogenic proteins linked to neurodegenerative diseases and is discussed as an early step in the formation of protein aggregates with neurotoxic properties. In prion diseases, neurodegeneration and formation of infectious prions is caused by aberrant folding of the cellular prion protein (PrP^C^). PrP^C^ is characterized by a large intrinsically disordered N-terminal domain and a structured C-terminal globular domain. A significant fraction of mature PrP^C^ is proteolytically processed *in vivo* into an entirely unstructured fragment, designated N1, and the corresponding C-terminal fragment C1 harboring the globular domain. Notably, N1 contains a polybasic motif that serves as a binding site for neurotoxic Aβ oligomers. PrP can undergo LLPS; however, nothing is known how phase separation of PrP is triggered on a molecular scale. Here, we show that the intrinsically disordered N1 domain is necessary and sufficient for LLPS of PrP. Similar to full-length PrP, the N1 fragment formed highly dynamic liquid-like droplets. Remarkably, a slightly shorter unstructured fragment, designated N2, which lacks the Aβ-binding domain and is generated under stress conditions, failed to form liquid-like droplets and instead formed amorphous assemblies of irregular structures. Through a mutational analysis, we identified three positively charged lysines in the postoctarepeat region as essential drivers of condensate formation, presumably largely *via* cation–π interactions. These findings provide insights into the molecular basis of LLPS of the mammalian prion protein and reveal a crucial role of the Aβ-binding domain in this process.

Various cellular processes occur in specialized compartments that can be membrane-surrounded organelles or biomolecular condensates formed by liquid–liquid phase separation (LLPS) (reviewed in (1, 2, 3)). On the molecular level, phase separation can be induced by intermolecular interactions between modular binding domains and cognate peptide motifs (4, 5). However, many phase-separating proteins contain large intrinsically disordered protein regions with low-complexity sequences, which do not form stable folded structures (6, 7). For those proteins, it has been described that multivalent (4, 8), that is, electrostatic (9, 10), π–π, cation–π (11, 12, 13, 14, 15), and hydrophobic (16) interactions can contribute to the formation of biomolecular condensates. Interestingly, many proteins implicated in neurodegenerative diseases have been shown to undergo LLPS, leading to the concept that altered LLPS can promote the formation of neurotoxic protein assemblies (reviewed in (17, 18, 19, 20)).

In mammals, a conformational transition of the cellular prion protein (PrP^C^) into the scrapie isoform leads to transmissible spongiform encephalopathies or prion diseases, such as Creutzfeldt–Jakob disease in humans, bovine spongiform encephalopathy in cattle, and scrapie in sheep and goat (21). PrP^C^ shows an interesting modular structure: an extended intrinsically disordered N-terminal domain and a highly structured C-terminal domain of a similar length (Fig. 1*A*). Various approaches in transgenic mice and cultured cells, including primary neurons, revealed that the N-terminal domain modulates signaling activity of PrP^C^. Strikingly, this role is seemingly contradictory because a physiological function of PrP^C^ to promote neuronal homeostasis under diverse stress conditions is linked to this domain as well as a pathophysiological activity of certain PrP mutants (reviewed in (22, 23, 24)). Moreover, neuronal PrP^C^ mediates neurotoxic effects of scrapie prions (25, 26, 27, 28), Aβ (29, 30, 31, 32, 33), α-synuclein (34), and Tau (35) *via* an interaction of its N-terminal domain with beta-sheet–rich oligomeric conformers of the respective pathogenic proteins. A considerable fraction of mature PrP^C^ is proteolytically processed (α-cleavage) under physiological conditions into a completely unstructured fragment, designated N1, and the corresponding C-terminal structured fragment C1. A second cleavage around amino acid position 90 (β-cleavage) is mainly observed under pathological conditions and generates the fragments N2 and C2 (36, 37, 38, 39) (Fig. 1*A*). Of note, N1 but not N2 contains the Aβ-binding domain (29, 40).

**Figure 1:**
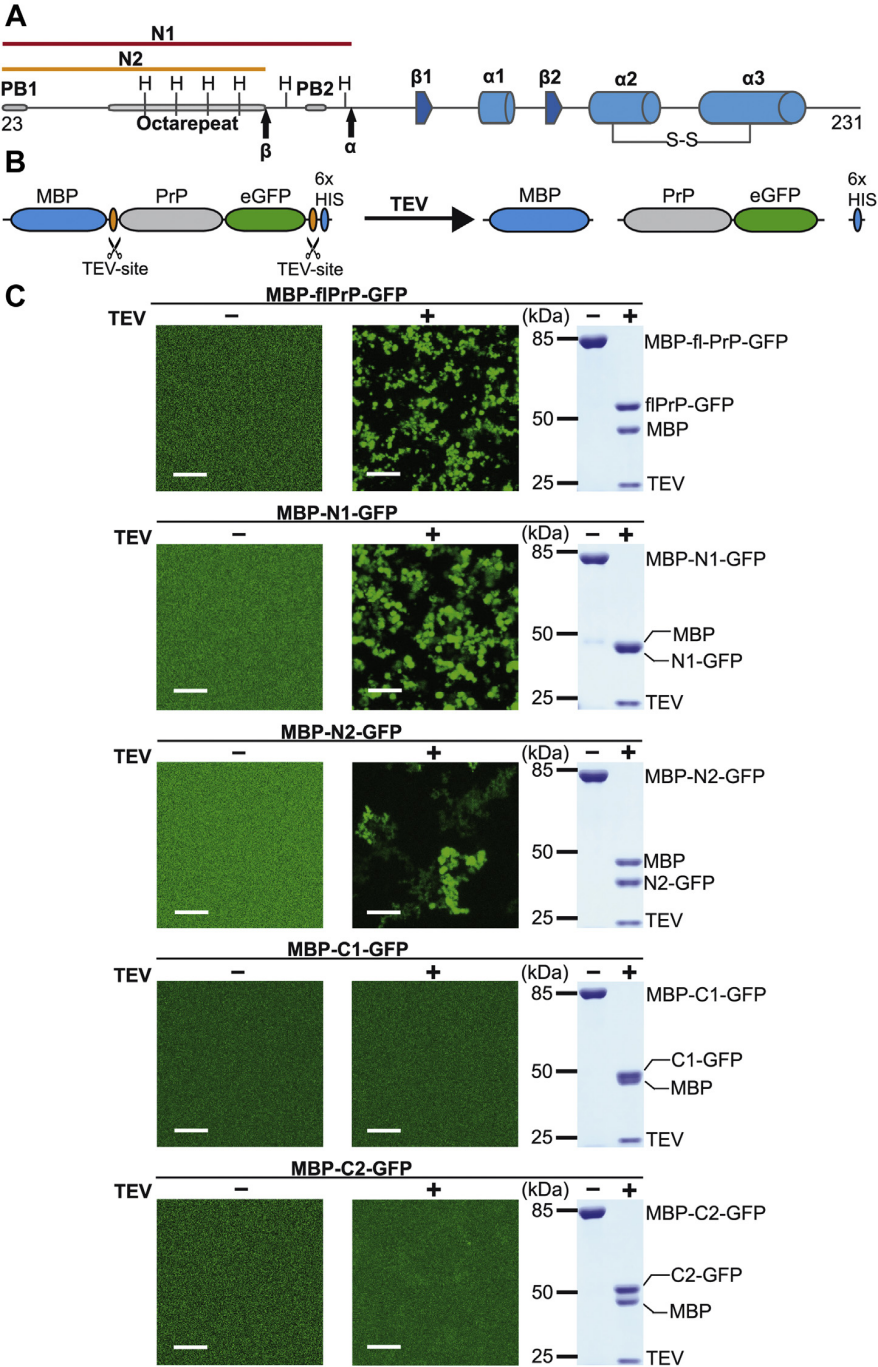
**The unstructured N1 fragment of PrP is necessary and sufficient for liquid–liquid phase separation.***A*, schematic representation of the mammalian prion protein. *B*, scheme of the experimental approach. The N-terminal maltose-binding protein (MBP) and the C-terminal His tag (6xHis) can be cut off from the PrP–GFP fusion protein by incubation with TEV protease. *C*, fusion proteins (30 μM in 10 mM Tris, pH 7.4) composed of full-length (fl) PrP or the indicated PrP fragments were incubated in the absence (TEV−) or presence (TEV+) of TEV protease for 1 h, and then, fluorescent image data were recorded by using a microscope. The scale bar represents 10 μm. An aliquot of each sample (4.5 μg) was analyzed in parallel by SDS-PAGE and Coomassie brilliant blue staining (*right panels*). The positions of the respective proteins are indicated. β, cleavage site that generates N2 and C2; α, cleavage site that generates N1 and C1; β1,2, beta strand; α1-3, alpha helices; H, histidine; PB, polybasic cluster; PrP, prion protein; S–S, disulfide bridge; TEV, tobacco etch virus.

The ability of full-length PrP to form biomolecular condensates has been described recently (41, 42, 43, 44, 45); however, the underlying molecular mechanisms that drive the formation of liquid-like droplets remain unknown. In the present study, we analyzed phase separation of full-length PrP and its major proteolytic fragments N1, N2, C1, and C2. Our experiments revealed that N1 is necessary and sufficient to drive phase separation of full-length PrP. Within N1, positively charged residues located in the Aβ-binding domain are essential for the formation of biomolecular condensates, most likely *via* cation–π interactions.

## Results and discussion

### N1–PrP but not N2–PrP undergoes LLPS

To study LLPS of PrP *in vitro*, we used maltose-binding protein (MBP)–PrP–eGFP fusion proteins (Fig. 1*B*). Phase separation was induced by tobacco etch virus (TEV) protease-mediated liberation of PrP–eGFP from MBP and monitored by laser scanning microscopy. Full-length PrP rapidly formed droplets upon TEV protease cleavage, indicative of LLPS (Fig. 1*C*, TEV+). A key feature of proteins undergoing condensate formation is the presence of intrinsically disordered and low-complexity regions (6), which are important drivers of phase separation. To study the role of the unstructured domain of PrP in LLPS, we cloned N1 and N2, the two disordered N-terminal fragments of PrP that are generated *in vivo* through proteolytic processing by an α- and β-cleavage, respectively (Fig. 1*A*). Indeed, N1 underwent phase separation similarly to full-length PrP, indicating that the structural elements in the C-terminal domain are not required for the formation of biomolecular condensates. In contrast, N2 formed irregular assemblies instead of liquid-like droplets (Fig. 1*C*, TEV+), which were not present before TEV cleavage (Fig. 1*C*, TEV−). These findings raised the question of whether the globular domain can undergo LLPS in the absence of the unstructured domains. Consequently, we analyzed C1 and C2, the C-terminal fragments generated after α- or β-cleavage, in parallel to N1 or N2 (Fig. 1*A*). Both before and after TEV cleavage, GFP fluorescence was evenly distributed, indicating that neither C1 nor C2 formed biomolecular condensates under these conditions, corroborating recent results (41). Of note, the lack of LLPS was not due to inefficient TEV cleavage (Fig. 1*C*, right panels).

**Figure 2:**
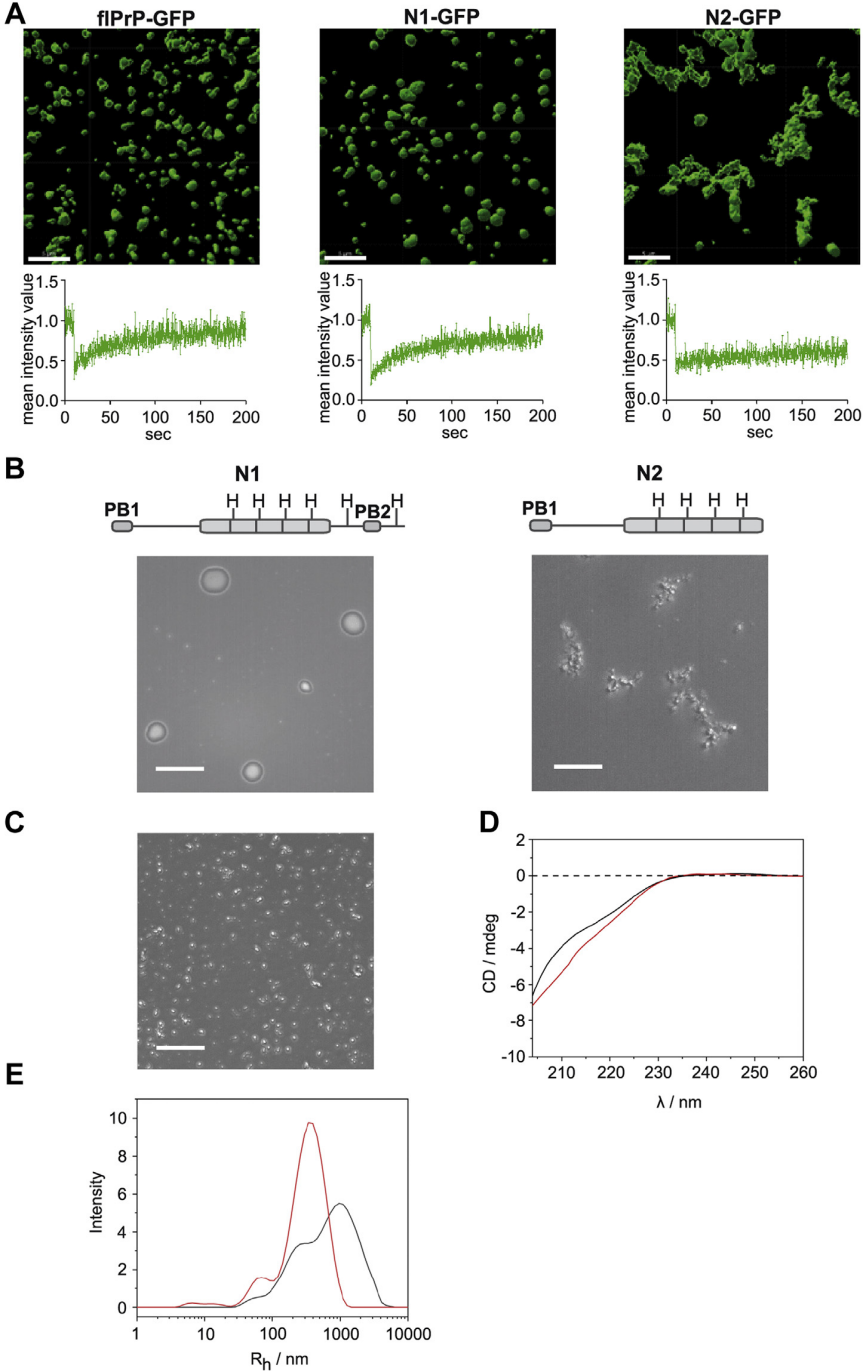
**Loss of the postoctarepeat domain interferes with the formation of condensates with liquid-like properties.***A*, volumetric three-dimensional reconstitution of the indicated proteins (10 μM in 10 mM Tris, pH 7.4) after TEV-mediated cleavage (1 h). Reconstitution was performed using Z-stack images (volume of 67.5 × 67.5 × 10 μm) produced by confocal laser scanning microscopy. The scale bar represents 5 μm (*upper panels*). Protein mobility within the droplets was measured by fluorescence recovery after photobleaching (FRAP). After 10 s of baseline recording (prebleach), a small area of interest (AOI) was photobleached. The average normalized fluorescence intensity of three AOIs was plotted over time (*lower panels*). *B*, MBP-N1 and MBP-N2 (10 μM in 10 mM Tris, pH 7.4) were analyzed by bright-field microscopy 1 h after TEV cleavage. A schematic representation of the fragments is shown above the images. *C* and *D*, recombinant N1 (64 μM in 20 mM Tris HCl, pH 7.4) was analyzed by bright-field microscopy (the scale bar represents 10 μm) (*C*) and CD spectroscopy (*D*). Shown are CD spectra in the absence (*black line*) and the presence of 4 M urea (*red line*). *E*, hydrodynamic radius (*R*_h_) distribution functions for a 50 μM solution of N1 in 20 mM Tris HCl, pH 7.4 (*black line*) and in a 4 M urea-containing buffer (*red line*). MBP, maltose-binding protein; TEV, tobacco etch virus.

To study the droplets/assemblies formed by N1 and N2 in more detail, we performed confocal laser scanning microscopy and recorded Z-stacks with a depth of 10 μm. Representative volumetric three-dimensional reconstructions supported the notion that N1 formed droplets, similarly to full-length PrPs, whereas the N2 assemblies were of irregular structures (Fig. 2*A*, upper panels). A signature of biomolecular condensates are highly dynamic molecules that rapidly exchange both within the condensate and with the surrounding environment. We recorded fluorescence recovery after photobleaching (FRAP) to test for a dynamic behavior of the PrP droplets. Full-length PrPs as well as N1 molecules in the droplets were highly dynamic, characteristic of a liquid-like state. In contrast, fluorescence recovery of the irregular N2 structures was greatly delayed, indicating formation of a gel-like or aggregated state (Fig. 2*A*, lower panels). Because we recorded FRAP 1 h after TEV protease-mediated cleavage, we considered the possibility that the N2 assemblies initially had liquid-like properties but then rapidly converted into a gel-like or aggregated state. Consequently, we did the FRAP analysis immediately after addition of TEV protease. Already under this condition, the N2 assemblies turned out to be nondynamic (Fig. S1*A*).

To address the possibility that the GFP tag influences LLPS, we purified MBP-N1 and MBP-N2 fusion proteins without GFP. Images taken by using bright-field microscopy confirmed that after TEV cleavage, MBP-N1 rapidly formed droplets, whereas MBP-N2 formed assemblies of irregular structures (Fig. 2*B*).

Next, we analyzed the conformation of phase-separated N1 after purifying recombinant N1 lacking both the MBP and GFP tag. N1(64 μM in 20 mM Tris HCl, pH 7.4) spontaneously formed liquid-like droplets, corroborating our findings with the TEV-induced cleavage of MBP-fusion proteins (Fig. 2*C*, Fig. S1*B*). At the same conditions, the CD spectrum of N1 is characterized by a negative CD band below 210 nm, indicating that the protein displays a fully disordered secondary structure at this condition (46). In the presence of 4 M urea, which prevents phase separation, only minor changes in the CD spectrum of N1 were observed. These findings show that the formation of liquid-like droplets by N1 is not accompanied by a significant conformational change (Fig. 2*D*). Finally, dynamic light scattering (DLS) experiments were carried out to determine the size of the N1 droplets (Fig. 2*E*). Based on the intensity distribution functions of the hydrodynamic radius, the N1 samples exhibited a largely polydisperse size distribution pattern, indicative of droplet formation of sizes ranging from 40 to several 1000 nm. Large hydrodynamic radius values beyond 1000 nm, that is, sizes in the μm range, indicate droplet formation as detectable by light microscopy. Interestingly, even in the urea-containing buffer, where macroscopic phase separation does not occur, N1 is not only present in its monomeric state but also forms larger oligomeric assemblies.

In sum, these experiments revealed that the unstructured domain of PrP is necessary and sufficient for LLPS. Furthermore, the comparative analysis of N1 and N2 has shown that the postoctarepeat region is crucial in driving LLPS of PrPs.

### The polybasic motif in the postoctarepeat region drives phase separation of PrPs

**Figure 3:**
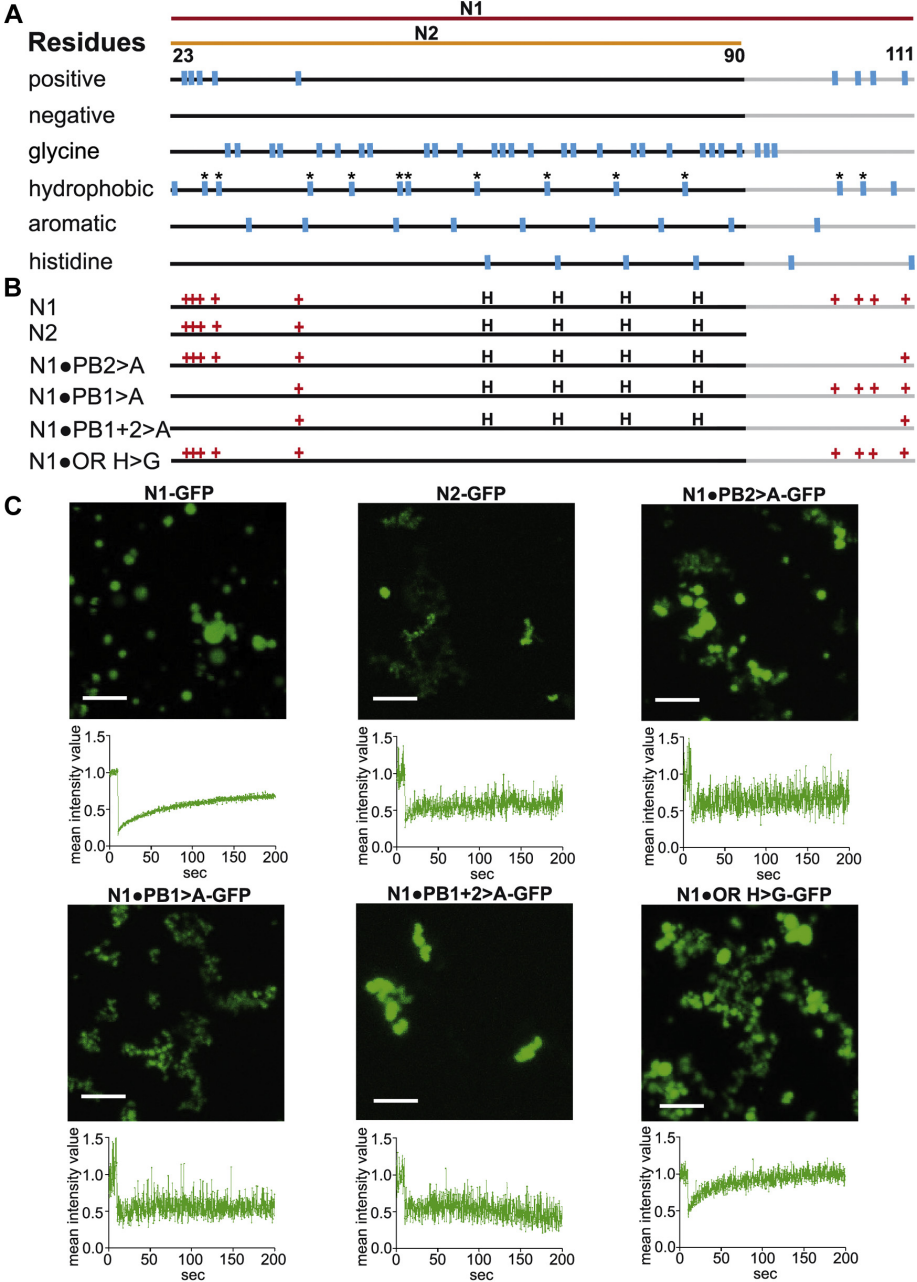
**The polybasic cluster of the Aβ-binding domain drives phase separation.***A*, schematic drawing of selected amino acids in the intrinsically disordered N-terminal domain. The domain that is missing from N2 (aa 90–111) is indicated in *gray*. *B*, schematic representation of the constructs. The positively charged residues (+) and the histidines (H) in the octarepeat domain are illustrated. *C*, the indicated proteins (10 μM in 10 mM Tris, pH 7.4) were analyzed 1 h after TEV cleavage. Fluorescent image data (*upper panels*) and FRAP (*lower panels*) were recorded as described in Figures 1 and 2. FRAP, fluorescence recovery after photobleaching; TEV, tobacco etch virus.

To gain insight into mechanisms underlying phase separation of N1, we had a closer look at amino acids that can drive phase separation *via* intermolecular interactions. The scheme in Figure 3*A* depicts the amino acid composition of the N-terminal domain of PrPs. The unstructured domain is devoid of any negatively charged amino acids and contains only nine amino acids with positively charged side chains, which are in either an N- or C-terminal cluster (PB1 and PB2; see also Fig. 1*A*). In the “sticker and spacer” model that is frequently used to describe LLPS by intrinsically disordered domains, glycine is generally considered a spacer (47). Thus, it is interesting that N1 contains numerous glycines. Hydrophobic residues are evenly distributed over the entire domain with all but two of them being prolines. This is worth mentioning based on a possible specific role they are playing in phase separation. The partially positively charged faces of the pyrrolidine ring has been described to mediate intermolecular interactions with the negatively charged π faces of aromatic residues (48). The aromatic amino acids are also rather evenly distributed within the N1 domain without obvious cluster formation. The location of the six histidine residues is indicated because copper binding to these histidines seems to influence the structure and physiological function of PrP^C^ as well as the formation of the scrapie isoform of prion protein (49, 50, 51, 52).

Comparing N2 with N1, the most striking difference is the lack of the polybasic cluster in the postoctarepeat domain (PB2), which serves as a binding domain for neurotoxic Aβ oligomers (29, 40). Conceptually, the positively charged lysines may contribute to phase separation *via* intermolecular cation–π interactions (11, 12, 13, 14). To test this hypothesis experimentally, we mutated the three lysines in PB2 to alanines (Fig. 3*B*) and analyzed phase separation. Indeed, the loss of the three lysine residues in the postoctarepeat region significantly impaired condensate formation of N1•PB2>A. The microscopic analysis revealed the formation of irregular structures, similarly to N2 (Fig. 3*C*, Figs. S2 and S3). Importantly, the FRAP recording supported the notion that N1•PB2>A lost its liquid-like properties. To support our concept that lysines are effective through their positive charge, we generated N1•PB2>R in which the three lysine residues in PB2 were mutated to positively charged arginines. Indeed, N1•PB2>R underwent LLPS similarly to N1 (Fig. S4A). In contrast, mutation of the aromatic residues (N1•W|Y > G) impaired LLPS, emphasizing the important role of cation–π interactions between positively charged residues and aromatic residues in driving LLPS of N1 (Fig. S4B). Based on these findings, we were wondering whether the other polybasic motif, located at the very N terminus, has a similar function in LLPS. N1•PB1>A did also not undergo LLPS and formed assemblies with irregular structures, indicating a gel-like or aggregated state, as judged by FRAP recordings (Fig. 3*C*, Figs. S2 and S3). As expected, deleting both the N- and C-terminal polybasic motifs (N1•PB1+2 > A) aggravated this phenotype. Finally, we mutated the four histidines within the octarepeat region. However, phase separation of N1•OR-H>G was not impaired (Fig. 3*C*, Figs. S2 and S3).

**Figure 4:**
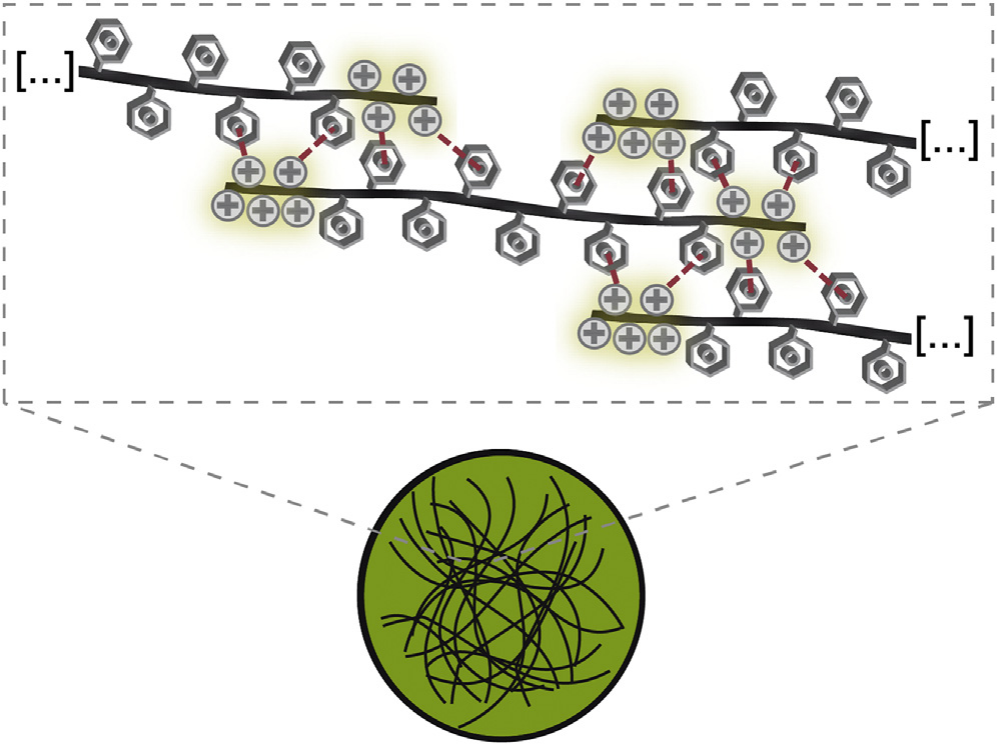
**Model for phase separation of N1.** In addition to favorable hydrophobic interactions, N1 mainly uses intermolecular cation–π interactions to form liquid droplets. Depicted are the aromatic rings of aromatic amino acid residues and the positive charge of lysine and arginine (+). *Red dashed lines* indicate the formation of cation–π interactions.

In conclusion, our study provided new mechanistic insights into the propensity of the mammalian prion protein to undergo LLPS. Our experiments revealed that the intrinsically disordered N1 fragment of PrP is necessary and sufficient for the formation of biomolecular condensates. On the molecular level, phase separation is governed primarily by basic residues within the Aβ-binding domain, most likely through intermolecular cation–π interactions of the lysines with neighboring aromatic side chains (Fig. 4). These findings are relevant for at least two major aspects in prion protein biology. First, binding of Aβ oligomers or other neurotoxic β-sheet–rich conformers to the polybasic motif is expected to interfere with LLPS of full-length PrPs and the N-terminal fragments, similarly to the deletion of the lysines. In support of this notion, it has been reported that binding of Aβ oligomers modulates phase separation and alters the conformation of full-length PrPs (44, 53). Second, it is tempting to speculate that LLPS of PrPs is linked to both its conversion into prions or neurotoxic conformers and its physiological function. Notably, this applies not only for the signaling competence of the full-length PrP^C^ but also for the biological activities of N1 after its liberation from the PrP^C^ by α-cleavage.

## Experimental procedures

### Constructs/plasmids

Plasmid maintenance and amplification was carried out using *Escherichia coli* TOP10 (Thermo Fisher Scientific). All PrP constructs were generated by standard PCR cloning techniques and are based on the coding region of mouse PrP gene (Prnp; GenBank accession number M18070) modified to express PrP-L108M/V111M (54), allowing detection by the mAb 3F4 (55). Full-length PrP: aa 23 to 230; N1: aa 23 to 114; N2: aa 23 to 89; C1: aa 111 to 230; C2: aa 90 to 230; N1•PB1>A: the polybasic motif K-K-R-P-K (aa 23–27) was changed to A-A-A-P-A; N1•PB2>A: the polybasic motif K-P-S-K-P-K (aa 100–105) was changed to A-P-S-A-P-A; N1•PB2>R: the polybasic motif K-P-S-K-P-K (aa 100–105) was changed to R-P-S-R-P-R; N1•PB1+2>A: both polybasic motifs were changed; N1•OR H>G: the four H in the octarepeat region (aa 49–89) were changed to G; N1•W|Y>G: all aromatic amino acid residues were changed to G. To generate the MBP-TEV-PrP-eGFP-TEV-His_6_ plasmids (Fig. 1*B*), we exchanged the FUS coding region from the pMal-TEV-FUS-eGFP-TEV-His_6_ plasmid, kindly provided by Dorothee Dormann (56), by those of the respective PrP variants.

### Protein expression and purification

MBP-PrP-eGFP constructs were transformed into either BL21-DE3 strains for N-terminal constructs or Origami B (DE3) competent cells (Novagen) for full-length PrP and C1 and C2 constructs. For protein expression, 1-l bacterial culture of the lysogeny broth medium was inoculated and grown to an absorbance (600 nm) of 0.9 to 1.0. For BL21-DE3 cultures, 30-min incubation on ice was performed before IPTG induction. Then, the expression was induced with 100 μM IPTG and the culture was incubated over night at 12 °C, 120 rpm. For Origami B (DE3) cultures, the expression was induced with 0.5 mM IPTG and incubated over night at 25 °C, 120 rpm. Bacteria were harvested by centrifugation (5,000*g*, 4 °C, 20 min), and the pellet was washed with 20 ml Millipore water and centrifuged again (2,000*g*, 4 °C, 20 min). Pellets were stored at −20 °C until further use. For purification, the bacterial pellet was resuspended in the lysis buffer (50 mM Na_2_HPO_4_/NaH_2_PO_4_ (pH 8.0), 500 mM NaCl, 0.01 mM ZnCl_2_, 10% glycerol). Protein lysis was performed *via* SLM AMINCO French Press (Thermo Fisher Scientific), and the protein solution was centrifuged (40,000*g*, 45 min, 4 °C). The supernatant was loaded on a His-Trap FF column (GE Healthcare) equilibrated with the lysis buffer and washed with three CV lysis buffer containing 20 mM imidazole and in case of the N-terminal constructs washed again with three CV lysis buffer containing 50 mM imidazole. Proteins were eluted with the lysis buffer containing 200 mM imidazole and dialyzed over night at 4 °C in the dialysis buffer (50 mM Na_2_HPO_4_/NaH_2_PO_4_ (pH 8.0), 500 mM NaCl, 0.01 mM ZnCl_2_, 5% glycerol). The protein concentration was determined by NanoDrop 2000 (Thermo Scientific) and aliquoted and stored at −80 °C until further use.

To study N1-PrP without an MBP tag, N1 was cloned into a pGATEV vector and expressed in and purified from *E. coli* BL21-DE3.

### Sample preparation

Protein aliquots were thawed on ice and centrifuged at 20,000*g* for 10 min at 4 °C to remove aggregates. Using Vivaspin 500 columns with 30-kDa molecular weight cut off (Sartorius Stedim biotech), the buffer was exchanged to 10 mM Tris, pH 7.4, by centrifuging five times for 7 min at 12,000*g* at 4 °C. After buffer exchange, the final protein concentration was again determined by NanoDrop 2000. For LLPS induction, TEV protease was added to the sample and incubated 1 h for complete cleavage before microscopy.

### Laser scanning microscopy

As described previously (57), fluorescent imaging laser scanning microscopy on a microscope (ELYRA PS.1; Carl Zeiss) with an imaging detector (LSM 880; Carl Zeiss) was performed. For z-stack scanning, a 63× numerical aperture 1.4 oil-immersion objective was used to record a stack of 67.5 × 67.5 × 10 μm and 0.330 μm for each optical section. The argon laser power was set to 0.006% at 488 nm with pixel dwell time of 5.71 μs. During all measurements, laser power, gain, and field of view were kept constant. Data were imported into Imaris 9.3.1 for three-dimensional analysis of the z-stack images, and the surface module was used for reconstruction of the surfaces. For FRAP experiments, ZEN2.1 bleaching and region software module and Plan-Apochromat 100× numerical aperture 1.46 oil differential interference contrast M27 objective was used. For regions of interest, three circular areas with a 12-pixel diameter were chosen. One region was bleached with 100% laser power and a pixel dwell time of 8.71 ms, with a scan time of 111.29 ms and a pixel dwell time of 1.61 ms, and the other two regions were used as the reference signal and background signal. Data calculation was performed in Excel 2016, and diagrams were made with GraphPad Prism.

### CD spectroscopy

CD spectroscopy experiments were carried out at the temperature of 25 °C by means of a Jasco J-715 spectropolarimeter from JASCO Corporation. Far-UV spectra of N1 were recorded at the concentration of 64 μM by using a quartz cuvette with a path length of 0.01 cm. The experiments were performed in 20 mM Tris HCl buffer, pH 7.4, in the absence and in the presence of 4 M urea. From each sample, an appropriate background spectrum was subtracted. All the experiments were performed with the following instrumental parameters: scan rate of 50 nm min^−1^, response time of 2 s, and bandwidth of 5 nm. It is important to note that owing to the strong absorbance of urea, it is not possible to record the CD spectra down to 190 nm.

### DLS

DLS measurements were performed by means of a Zetasizer Nano S from Malvern Instruments Limited. The instrument is equipped with a He–Ne laser operating at 633 nm. Each size distribution function is the result of 15 accumulations. The concentration of the N1 sample was 50 μM. The experiments were performed in 20 mM Tris HCl buffer, pH 7.4, in the absence and in the presence of 4 M urea at the temperature of 25 °C.

## Data availability

All data are contained within the article and the supporting information.

## Supporting information

This article contains supporting information.

## Conflict of interest

The authors declare that they have no conflicts of interest with the contents of this article.
