# Supplementary material for: The N-terminal domain of the prion protein is required and sufficient for liquid–liquid phase separation: A crucial role of the Aβ-binding domain
Source: J Biol Chem. 2021 Jun 6;297(1):100860. doi: 10.1016/j.jbc.2021.100860 (PMC8254114; doi:10.1016/j.jbc.2021.100860)
Supplement: Supplemental Figures S1–S4 [file mmc1.pdf]

Supporting Information

The N-terminal domain of the prion protein is required and sufficient for liquid-liquid phase separation; a crucial role of the Aβ-binding domain

Janine Kamps[1, 2], Yu-Hsuan Lin[1], Rosario Oliva[3], Verian Bader[4], Roland Winter[2, 3], Konstanze F. Winklhofer[2, 4] and Jörg Tatzelt[1,2#]

[1]Department Biochemistry of Neurodegenerative Diseases, Institute of Biochemistry and Pathobiochemistry, Ruhr University Bochum, Germany; [2]Cluster of Excellence RESOLV, Bochum, Germany, [3]Physical Chemistry I - Biophysical Chemistry, Faculty of Chemistry and Chemical Biology, TU Dortmund University, Dortmund, Germany, [4]Department Molecular Cell Biology, Institute of Biochemistry and Pathobiochemistry, Ruhr University Bochum, Germany

Running title: Liquid-liquid phase separation of the prion protein

# To whom correspondence should be addressed: Jörg Tatzelt, Ruhr University Bochum, Universitätsstr. 150, D-44801 Bochum, Germany; phone: +49 234 32-22429; fax: +49 234 32-14193; e-mail: Joerg.Tatzelt@rub.de

Keywords: liquid-liquid phase separation; neurodegenerative disease; prion protein; prion disease; protein self-assembly; aggregation; intrinsically disordered protein

Supplement Figure 1

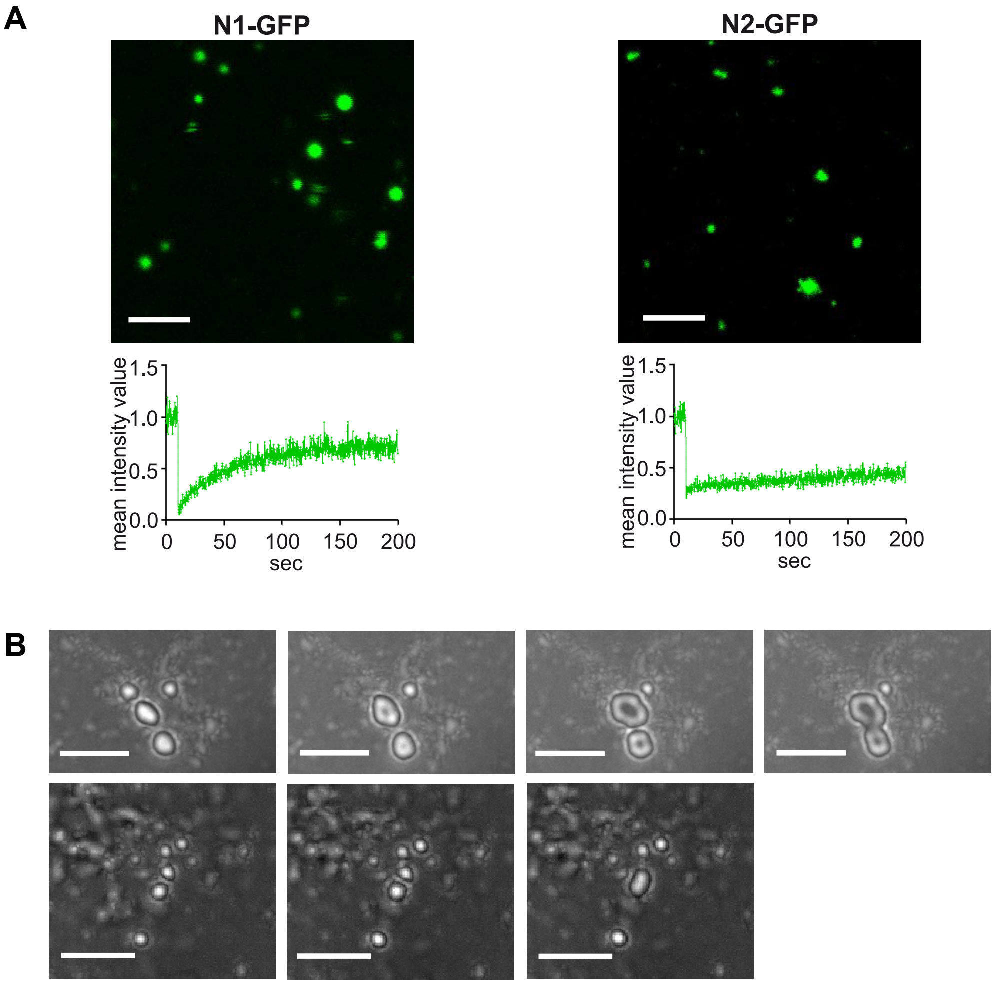

Figure S1. (A) N2-GFP forms non-dynamic assemblies directly after TEV cleavage. The fluorescent image data of MBP-N1-GFP and MBP-N2-GFP (10 µM in 10 mM Tris pH 7.4, upper panels) were directly recorded on a microscope after addition of TEV protease (scale bar, 10 µm). FRAP data (lower panels) were recorded as described in Figs 1 and 2. **(B) MBP-N1 undergoes fusion.** MBP-N1 (10 µM in 10 mM Tris pH 7.4) was analyzed by bright-field microscopy 2 h after TEV cleavage (scale bar, 5 µm). Shown are two different fusion events (upper and lower lane). The pictures were taken every 30 seconds over a period of 90 seconds (upper panel) or 60 seconds (lower panel).

Supplement Figure 2

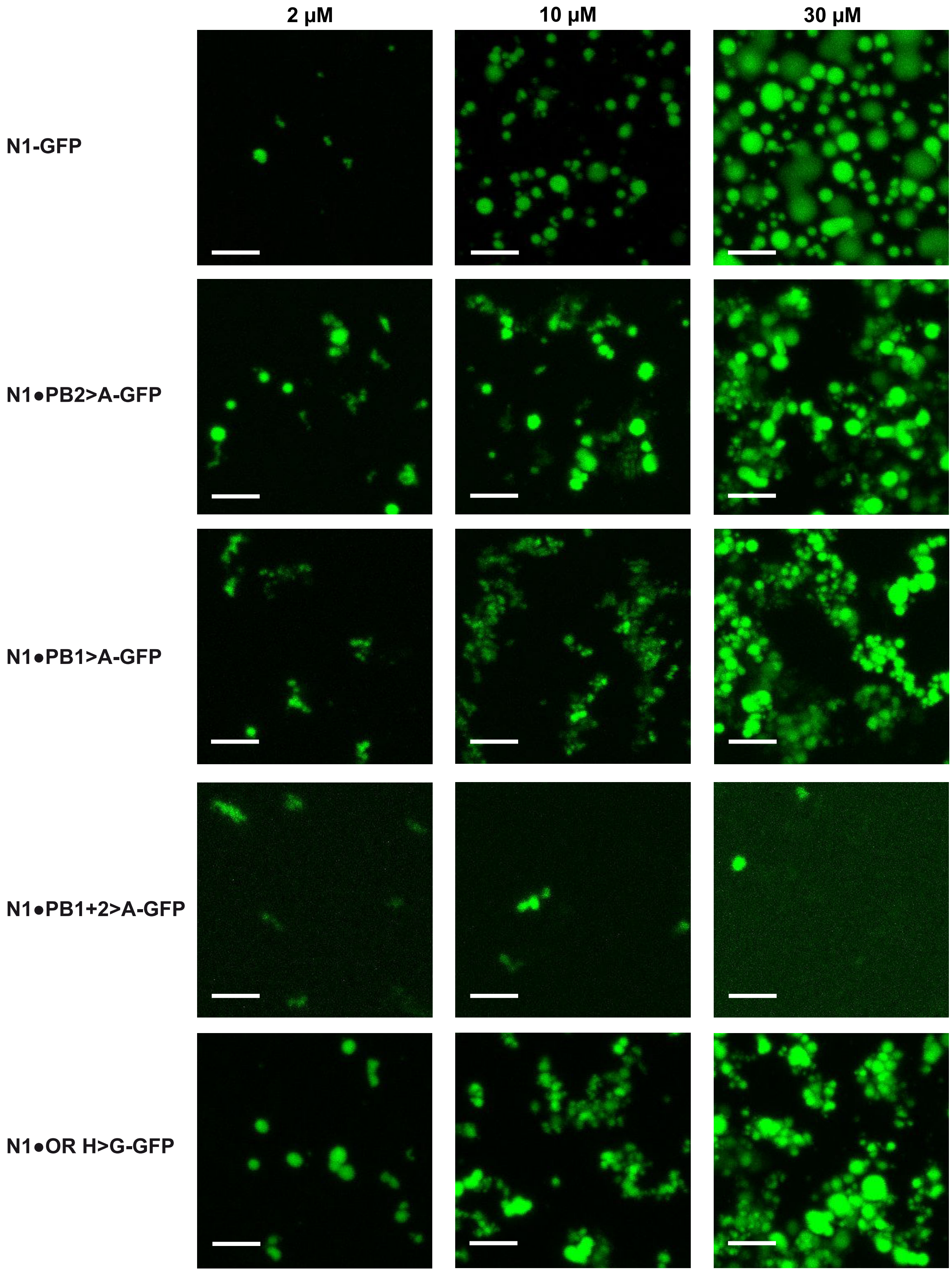

Figure S2. The indicated proteins were incubated in the presence of TEV protease for 1h and then fluorescent image data were recorded on a microscope (scale bar, 10 µm).

Supplement Figure 3

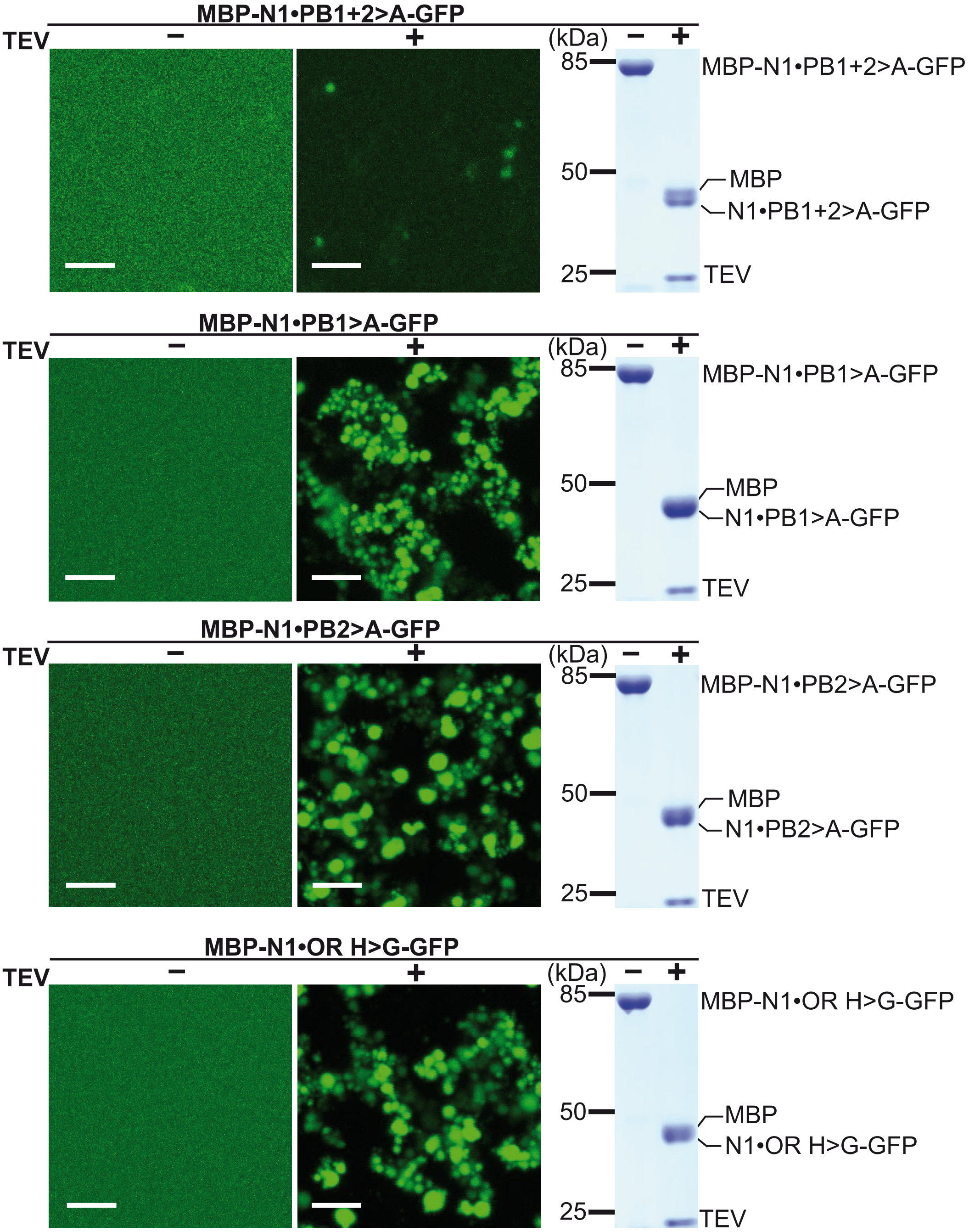

Figure S3. Mutations in the polybasic regions interfere with LLPS. PrP variants were incubated in the absence (TEV -) or presence of TEV protease (TEV +) for 1h and then fluorescent image data were recorded on a microscope (scale bar, 10 µm). An aliquot of each sample (4.5 µg) was analyzed in parallel by SDS-PAGE and Coomassie brilliant blue staining (right panels). The respective proteins are indicated.

Supplement Figure 4

A **MBP-N1•PB2>R-GFP**

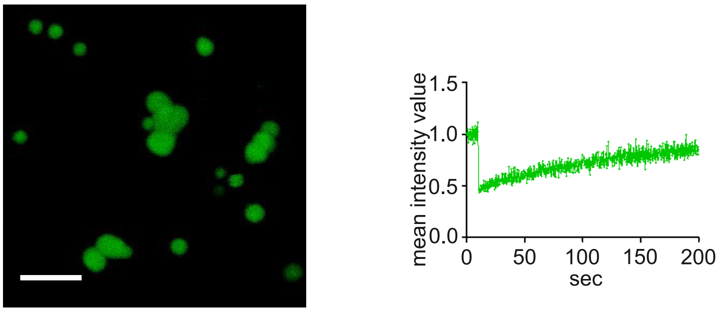

B **MBP-N1•W│Y>G-GFP**

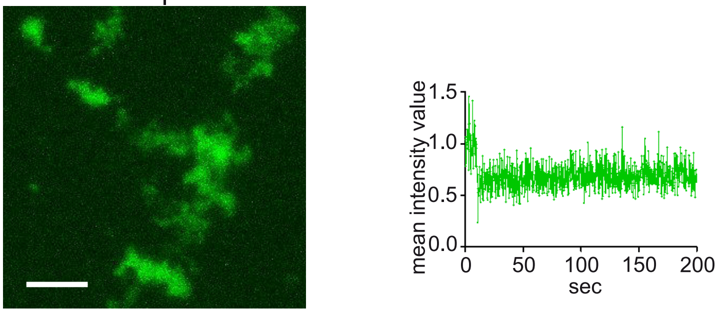

Figure S4. (A) MBP-N1•PB2>R-GFP undergoes liquid-liquid phase separation. (B) Impaired LLPS of MBP-N1•W│Y>G-GFP. The indicated proteins (10 μM in 10 mM Tris pH 7.4) were analyzed 1 h after TEV cleavage. Fluorescent image data (left panels) and FRAP (right panels) were recorded as described in Figs 1 and 2 (scale bar, 10 μm).
